# Maternal exposure to combustion generated PM inhibits pulmonary Th1 maturation and concomitantly enhances postnatal asthma development in offspring

**DOI:** 10.1186/1743-8977-10-29

**Published:** 2013-07-16

**Authors:** Pingli Wang, Dahui You, Jordy Saravia, Huahao Shen, Stephania A Cormier

**Affiliations:** 1 Department of Pediatrics, University of Tennessee Health Sciences Center, 50 North Dunlap Street, Room 416R, Memphis, Tennessee 38013, USA; 2 Department of Respiratory Disease, Second Affiliated Hospital, Zhejiang University School of Medicine, Hangzhou, China; 3 State Key Lab of Respiratory Disease, Guangzhou, China

**Keywords:** Maternal exposure, Particulate matter, Offspring, Asthma

## Abstract

**Background:**

Epidemiological studies suggest that maternal exposure to environmental hazards, such as particulate matter, is associated with increased incidence of asthma in childhood. We hypothesized that maternal exposure to combustion derived ultrafine particles containing persistent free radicals (MCP230) disrupts the development of the infant immune system and results in aberrant immune responses to allergens and enhances asthma severity.

**Methods:**

Pregnant C57/BL6 mice received MCP230 or saline by oropharyngeal aspiration on gestational days 10 and 17. Three days after the second administration, blood was collected from MCP230 or saline treated dams and 8-isoprostanes in the serum were measured to assess maternal oxidative stress. Pulmonary T cell populations were assayed in the infant mice at six days, three and six weeks of postnatal age. When the infant mice matured to adults (i.e. six weeks of age), an asthma model was established with ovalbumin (OVA). Airway inflammation, mucus production and airway hyperresponsiveness were then examined.

**Results:**

Maternal exposure to MCP230 induced systemic oxidative stress. The development of pulmonary T helper (Th1/Th2/Th17) and T regulatory (Treg) cells were inhibited in the infant offspring from MCP230-exposed dams. As the offspring matured, the development of Th2 and Treg cells recovered and eventually became equivalent to that of offspring from non-exposed dams. However, Th1 and Th17 cells remained attenuated through 6 weeks of age. Following OVA sensitization and challenge, mice from MCP230-exposed dams exhibited greater airway hyperresponsiveness, eosinophilia and pulmonary Th2 responses compared to offspring from non-exposed dams.

**Conclusions:**

Our data suggest that maternal exposure to MCP230 enhances postnatal asthma development in mice, which might be related to the inhibition of pulmonary Th1 maturation and systemic oxidative stress in the dams.

## Background

Asthma is one of the most common chronic lung diseases in the world. The prevalence of asthma has risen in the past few decades, currently affecting one in ten children [1]. Epidemiological studies suggest that maternal stress during pregnancy and exposure of infants to environmental hazards, such as combustion generated particulate matter (PM) including tobacco smoke, is associated with the increased incidence of asthma [2-8]. Despite the evidence associating early PM exposure with asthma prevalence, there is little research on this subject and the mechanisms underlying this phenomenon are largely unknown.

A balanced and fully functional immune system is important to combat the numerous diseases of childhood and adult life. Reports stress that early-life exposure to xenobiotics poses the greatest environmental risk for the immune system and would be expected to exert the greatest effect on subsequent human health and diseases, such as asthma [9]. Disruption of immune development by means of maternal/perinatal exposure to immunotoxic agents might result in reduced postnatal immune responses or, conversely, exacerbate postnatal aberrant immune responses. Limited information is available about PM altering the immune system during the perinatal time period.

PM from waste combustion is an important portion of environmental air pollution. In this study, we used environmentally-relevant model ultrafine particle containing persistent free radicals (EPFR-UFP). These model EPFR-UFP contain 2-monochlorophenol (2-MCP), common by-product of waste combustion, chemisorbed to a transition metal-containing fly-ash. EPFR-UFP exist, therefore as a particle-pollutant system until a subsequent chemical reaction separates them [10]. We are specifically studying the EPFR of 2-MCP that is formed by reaction with Cu(II)O containing fly-ash at 230°C (referred to as MCP230) [11-13]. Our previous studies demonstrated that MCP230 exposure in adult mice induced a Th17-biased immune response, and increased neutrophilia inflammation in the lungs following induction of allergic asthma [14]. Given the differences between the fetal and adult immune systems, we hypothesized that exposure to EPFR-UFP at these different ages would elicit different immunological responses and alter the development of asthma. In this study, we examined the effects of maternal exposure to EPFR-UFP on maturational changes of the pulmonary immune response and postnatal allergen-induced asthma development in the offspring.

## Results

### Effect of MCP230 exposure on the pulmonary T cell response during postnatal development

**Figure 1:**
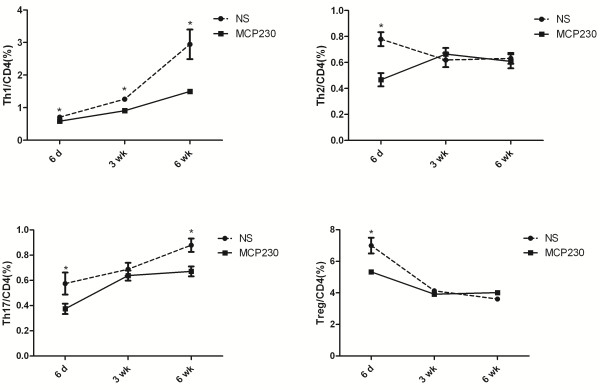
**The effects of maternal exposure to MCP230 on the pulmonary T cell populations in postnatal life of offspring.** Lung samples were obtained from infant mice at six days, three weeks and six weeks of postnatal age. Lung cells were isolated, stimulated with PMA and ionomycin, and stained with surface antibodies and intracellular antibodies. T cells subsets were then quantified using flow cytometry. Data were expressed as means ± SEM (*n* =5-8/group). *p < 0.05, NS vs. MCP230.

To examine the effect of maternal MCP230 exposure on the pulmonary T cell response in postnatal life, lung samples were taken at six days, three weeks and six weeks of age, and the T cell populations in lungs were analyzed by flow cytometry. As shown in Figure 1, offspring of dams exposed to MCP230 exhibited a significant reduction in the total proportion of pulmonary T helper (Th1/Th2/Th17) and CD4 + CD25 + Foxp3+ regulatory T (Treg) cells at 6 days of age. As the offspring of NS mice matured, the percentage of Th1 and Th17 cells increased, while the percentage of Th2 and Treg decreased. As the offspring of the MCP230-exposed mice matured, the percentage of Th1 cells barely increased and at 6 weeks of age remained diminished compared with offspring of NS mice. The percentage of Th17 cells followed a similar trend and remained diminished at 6 weeks old compared with offspring of NS mice. Interestingly, the percentage of Th2 and Treg cells returned to control levels at 3 weeks of age and remained equivalent to offspring of NS mice at 6 weeks of age.

### Maternal exposure to MCP230 increased allergen-induced airway hyperresponsiveness (AHR) in adult offspring

**Figure 2:**
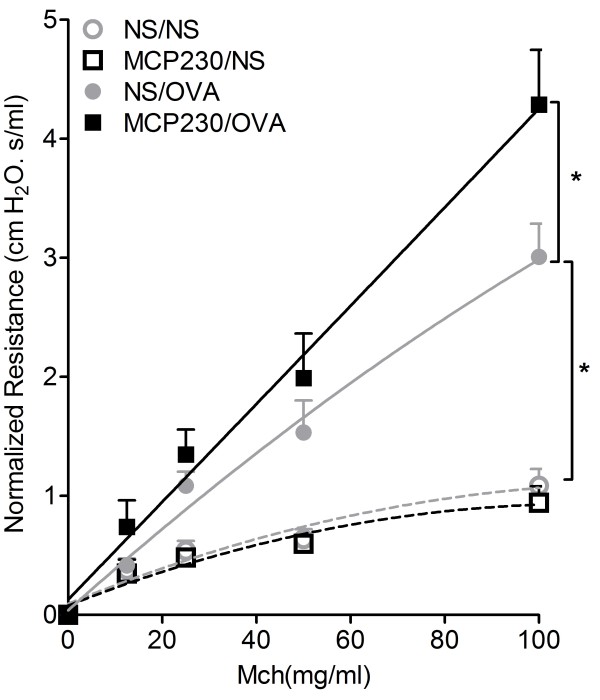
**The effect of maternal exposure to MCP230 on the development of AHR in offspring.** AHR to Mch was assessed 48 h after the final aerosol challenge. Results are expressed as airway resistance in mice exposed to nebulized Mch. Individual responses were normalized to their perspecitve baseline (0 mg/ml Mch) and are expressed as normalized means ± SEM (*n* =6-10/group). *p < 0.05.

Since maternal exposure to MCP230 suppressed pulmonary Th1 maturation, we were curious to know how this affected asthma development in the offspring. When the offspring of exposed dams mature to 6 weeks of age, an asthma model was established by OVA sensitization and challenge. Figure 2 shows the effect of maternal exposure to MCP230 on the development of asthmatic AHR in the offspring. There was no difference in baseline resistance between the MCP230-exposed and NS group (0.76 ± 0.03 vs 0.70 ± 0.05 cmH_2_O.s/ml, MCP230/NS vs NS/NS). AHR to methacholine (Mch) was also similar between the MCP230/NS and NS/NS groups. AHR to Mch was significantly increased in MCP230/OVA mice compared with mice in the NS/OVA group (*p* < 0.05).

### Following allergen challenge pulmonary eosinophilia and mucous production was greater in offspring of MCP230 exposed dams

**Figure 3:**
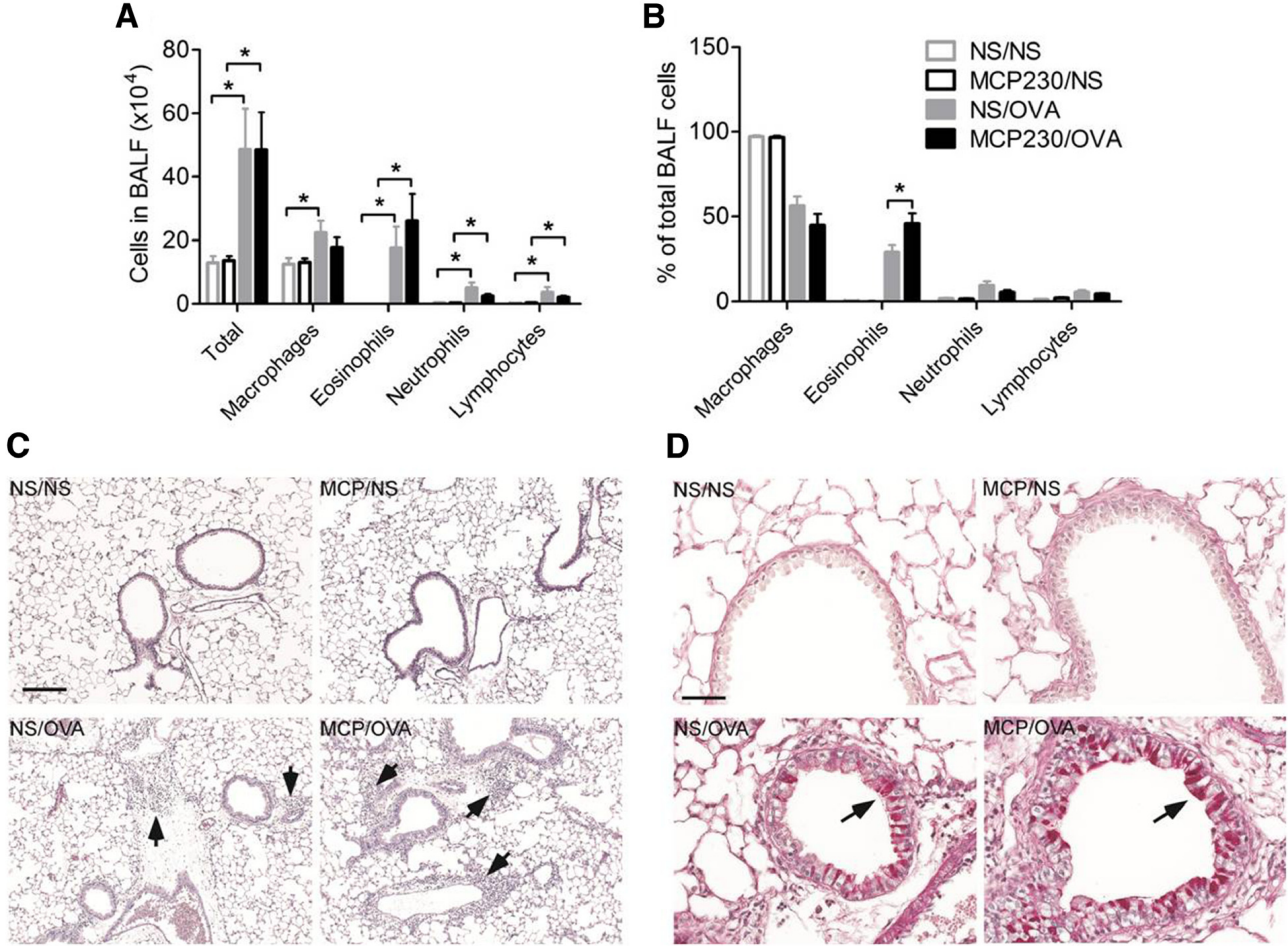
**Effect of maternal MCP230 exposure on pulmonary inflammation and mucus production in offspring after induction of allergic asthma.** The mouse model of asthma was established by OVA sensitization and challenge beginning at six weeks of age. **(A/B)** BALF was taken and cells were stained and differentiated and counted. Data were expressed as means ± SEM (n =8-10/group). *p < 0.05. Lungs of mice exposed to NS/NS, MCP230/NS, NS/OVA, or MCP230/OVA were removed, fixed, sectioned and stained with **(C)** H&E and **(D)** PAS. Scale bar = 50 μm **(C)** or 200 μm **(D)**. Arrows point to inflammatory cells **(C)** or mucus producing cells **(D)**.

To determine whether maternal MCP230 exposure alters allergen-induced pulmonary inflammation, we quantified total and differential cell counts in bronchoalveolar lavage fluid (BALF). We found no significant difference in the total cell number between the MCP230/OVA and NS/OVA groups (Figure 3A). But there was a significant increase in the percentage of eosinophils in BALF from MCP230/OVA mice (Figure 3B) compared with NS/OVA mice. Lung histopathology also showed more eosinophils in the lungs of MCP230/OVA mice than NS/OVA mice (Figure 3C). PAS staining demonstrated widespread mucus production in the bronchioles of MCP230/OVA mice and more mucus producing cells were observed in the lung of MCP230/OVA mice compared to NS/OVA mice (48.33 ± 5.17 vs. 28.44 ± 3.83 positive cells/mm basement membrane; Figure 3D). No obvious inflammatory cells infiltration or mucus production was observed in either the MCP230/NS or the NS/NS group of mice.

### Maternal exposure to MCP230 altered pulmonary Th cell subsets and cytokine responses in the offspring after induction of the asthma model

**Figure 4:**
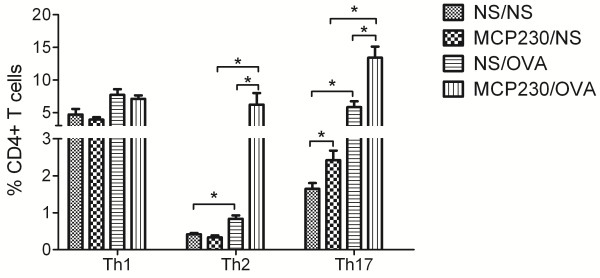
**Effect of maternal MCP230 exposure on pulmonary Th cell subsets in the offspring after induction of allergic asthma (OVA model).** Forty-eight h after the final aerosol OVA challenge, lung cells were isolated and T cells subsets were quantified using flow cytometry. Data were expressed as means ± SEM (n =5-6/group). *p < 0.05.

We further examined the Th subsets in the lungs of asthmatic mice. As shown in Figure 4, there were no significant differences in the levels of Th1 and Th2 cells in the MCP230/NS and NS/NS groups, but higher percentages of Th17 were observed in the offspring the MCP230/NS as compared to the NS/NS group. MCP230/OVA mice exhibited higher percentages of Th2 and Th17 cells than NS/OVA mice. The percentage of Th1 cells was comparable between the MCP230/OVA and the NS/OVA groups.

The cytokines in BALF were also analyzed. A total of 10 cytokines and chemokines (IFN-γ, IL-4, IL-5, IL-13, IL-17A, IL-10, RANTES, KC, MCP-1, MIP-1α) were measured. Consistent with our flow data, we observed elevated levels of IL-4, IL-5, and RANTES (Table 1) in the BALF of MCP230/OVA mice compared to NS/OVA mice (p < 0.05). The BALF levels of IFN-γ, IL-10, IL-13, KC and MCP-1 were comparable among these two groups. The levels of IL-17A and MIP-1α were below the limit of sensitivity of the assay in all groups.

**Table 1:** Cytokines and chemokines in BALF (pg/ml)

	**NS/NS**	**MCP230/NS**	**NS/OVA**	**MCP230/OVA**
IFN-γ	11.17 ± 5.26	6.69 ± 2.00	12.77 ± 1.08	10.73 ± 2.75
IL-4	N.D.	N.D.	9.22 ± 1.26	18.62 ± 3.48 #
IL-5	N.D.	N.D.	28.09 ± 10.96	38.59 ± 16.26 #
IL-13	5.69 ± 1.79	2.46 ± 0.98	12.36 ± 2.85*	20.91 ± 4.27*
IL-10	3.27 ± 0.74	1.63 ± 0.30	2.89 ± 0.28	4.08 ± 0.46*
RANTES	N.D.	N.D.	1.46 ± 0.12	2.19 ± 0.20 #
MCP-1	N.D.	N.D.	23.03 ± 4.84	26.08 ± 7.66
KC	9.82 ± 0.68	9.24 ± 0.28	16.00 ± 1.48*	15.11 ± 1.02*

Cytokines and chemokines in BALF were examined using a Milliplex mouse cytokine/chemokine assay kit. Results are expressed as means ± SEM. n = 7-8 mice/group. #*p* < 0.05 MCP230/OVA vs. NS/OVA; *p < 0.05 NS/OVA vs. NS/NS, MCP230/OVA vs. MCP230/NS; N.D.: non-detected.

### Maternal exposure to MCP230 induced systemic oxidative stress in dams

**Figure 5:**
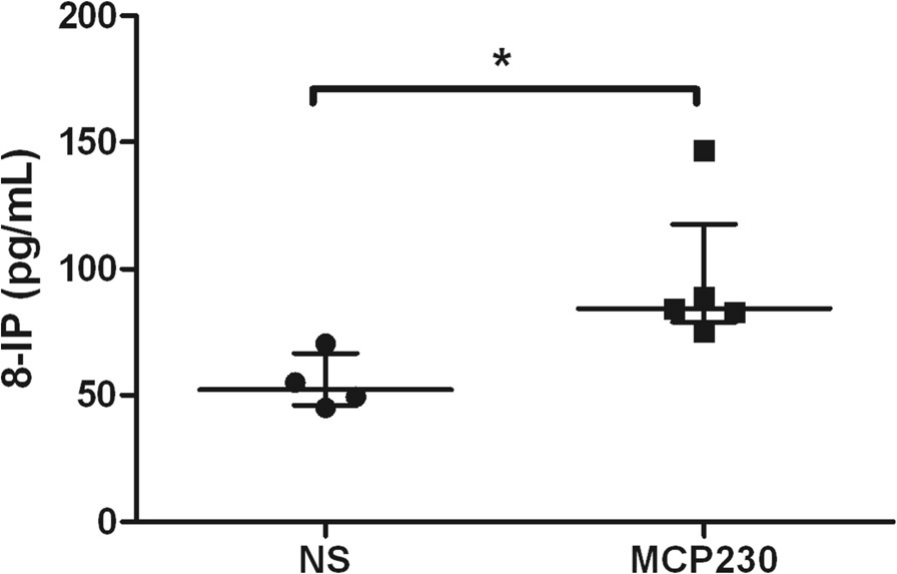
**MCP230 exposure induced oxidative stress in dams.** Blood was collected from dams 3 days after the second exposure to MCP230 or saline, and 8-IP was measured in the serum. Data were expressed as means ± SEM (*n* =4-5/group). *p < 0.05.

It is unlikely that a significant amount of MCP230 reached the developing fetus or fetal lung, therefore we hypothesized that systemic oxidative stress in the dam induced upon exposure to MCP230 was at least partly responsible for altering the pulmonary immune profile and exacerbating allergen-induced asthma development. To examine if exposure to MCP230, an environmentally persistent free radical, induced oxidative stress in the dams, we measured the level of 8-isoprostanes (8-IP), a sensitive index for oxidative stress [15], in the dam’s serum at 3 days after the second exposure to MCP230. As shown in Figure 5, we observed higher level of 8-IP in the serum of dams exposed to MCP230 compared to that of saline exposed dams (95.5 ± 12.99 vs 54.95 ± 5.54 pg/ml). These data demonstrate that exposure to MCP230 induced oxidative stress in the dams and the presence of maternal oxidative stress correlated to alterations in the pulmonary immune profile.

## Discussion

This manuscript explores the effects of maternal exposure to airborne PM on postnatal asthma development. Our data demonstrated that maternal exposure to MCP230 induced oxidative stress in the dams and enhanced allergen-induced asthma pathophysiology in the offspring, including increased severity of pulmonary eosinophilic inflammation, AHR in response to methacholine, and Th2 cell percentages and cytokine levels in BALF (e.g. IL-4 and IL-5). These findings are in stark contrast to our previous research of MCP230 exposure in adult mice, which was characterized by increased neutrophilic inflammation but not eosinophilic inflammation [14]. In addition, MCP230 exposure in adult mice induced higher Th17, but not Th2 immune response in lungs. To understand this difference, we studied the effects of maternal MCP230 exposure on the pulmonary immune development in infants.

Since CD4+ T cells play a pivotal role in asthma, in this study we focused on the development of CD4+ T subsets with age following maternal PM exposure. We found that the percentage of Th1 and Th17 in lungs increased progressively with age in mice; however the percentage of Th2 and Treg decreased during the first three weeks of postnatal life, which is consistent with human or primate subjects [16,17]. Maternal exposure to MCP230 stifled pulmonary T helper (Th1/Th2/Th17) and CD4 + CD25 + Foxp3+ T regulatory cell numbers in early life (<one week old). As the mice matured, Th2 and Treg responses recovered and eventually became equivalent to control mice. However, Th1 responses remained suppressed even at six weeks of age compared with control mice. This failure to increase Th1 cell numbers while increasing Th2 cell numbers might predispose the individual to Th2 dominated immune responses following allergen exposure and predisposes them to asthma. Clinical data demonstrated that allergic responses in children are most prevalent among those who previously developed attenuated Th1 responses during infancy [18].

The mechanisms by which maternal PM exposure alters Th immune development remain unknown. Some research suggests that epigenetic mechanisms, including DNA methylation, histone modifications, and microRNA (miRNA) expression, mediate toxicity from environmental pollutants [19,20]. Most studies conducted so far pinpoint a role for DNA methylation, which is usually associated with decreased expression of the gene (gene silencing) [21]. In fact, several recent studies demonstrate that exposure to chemical toxicants induces suppression of fetal IFN-γ production via hypermethylation of the IFN-γ gene promoter in CD4 + T cells [22-24], and thus, alters the Th immune response. In addition to hypermethylation, oxidative stress also interferes with the ability of methyltransferases to interact with DNA [25] and results in generalized methylation of cytosine residues at CpG sites and thus suppression of IFN-γ production. Other studies confirmed that in an oxidative environment, the ability of Th1 cells to expand and is also decreased [26,27]. Our previous studies demonstrated that MCP230 was a strong inducer of oxidative stress [13,14]. In this study, we found that MCP230 exposure induced oxidative stress in the serum of pregnant mice (Figure 5), and therefore demonstrated that systemic oxidative stress in dams correlated to inhibition of the development of pulmonary immune cells in offspring.

As the prenatal period is a time of extreme sensitivity to toxicant exposure. Postnatal consequences from such exposures during the initial establishment of the immune system and its maturation may be both more severe and more persistent than those that occur in adult animals exposed at similar or greater levels [9]. In the current study, we found that maternal exposure to MCP230 altered homeostatic baseline Th profiles. In particular, we observed reduced pulmonary Th1 and Th17cells through six weeks of age. Although at ten weeks of age Th1 cell numbers from offspring of exposed dams became equivalent to those of offspring from non-exposed dams, Th17 cells actually exceeded those of offspring from non-exposed dams (compare Figures 1 and 4). These data suggest that maternal exposure to MCP230 delays Th1 and Th17 maturation creating a window of enhanced vulnerability to infections and even asthma development, but these effects are not permanent.

## Conclusion

In summary, our mouse data recapitulate epidemiological data that indicate an association between maternal exposure to PM and asthma development in childhood. We further demonstrated that this enhancement in asthma development in offspring is associated with exposure induced systemic oxidative stress in dams and inhibition of pulmonary Th1 maturation in offspring.

## Materials and methods

### Animal protocols

C57/BL6 mice were purchased as breeders from Harlan (Indianapolis, IN), and maintained under specific pathogen-free conditions in the Louisiana State University Health Sciences Center vivarium. Breeders were time-mated, and the dams and their progeny were used for experiments. All animal protocols were prepared in accordance with the Guide for the Care and Use of Laboratory Animals [28] and approved by the Institutional Animal Care and Use Committee at Louisiana State University Health Sciences Center (New Orleans, LA).

### MCP230 exposure

**Figure 6:**
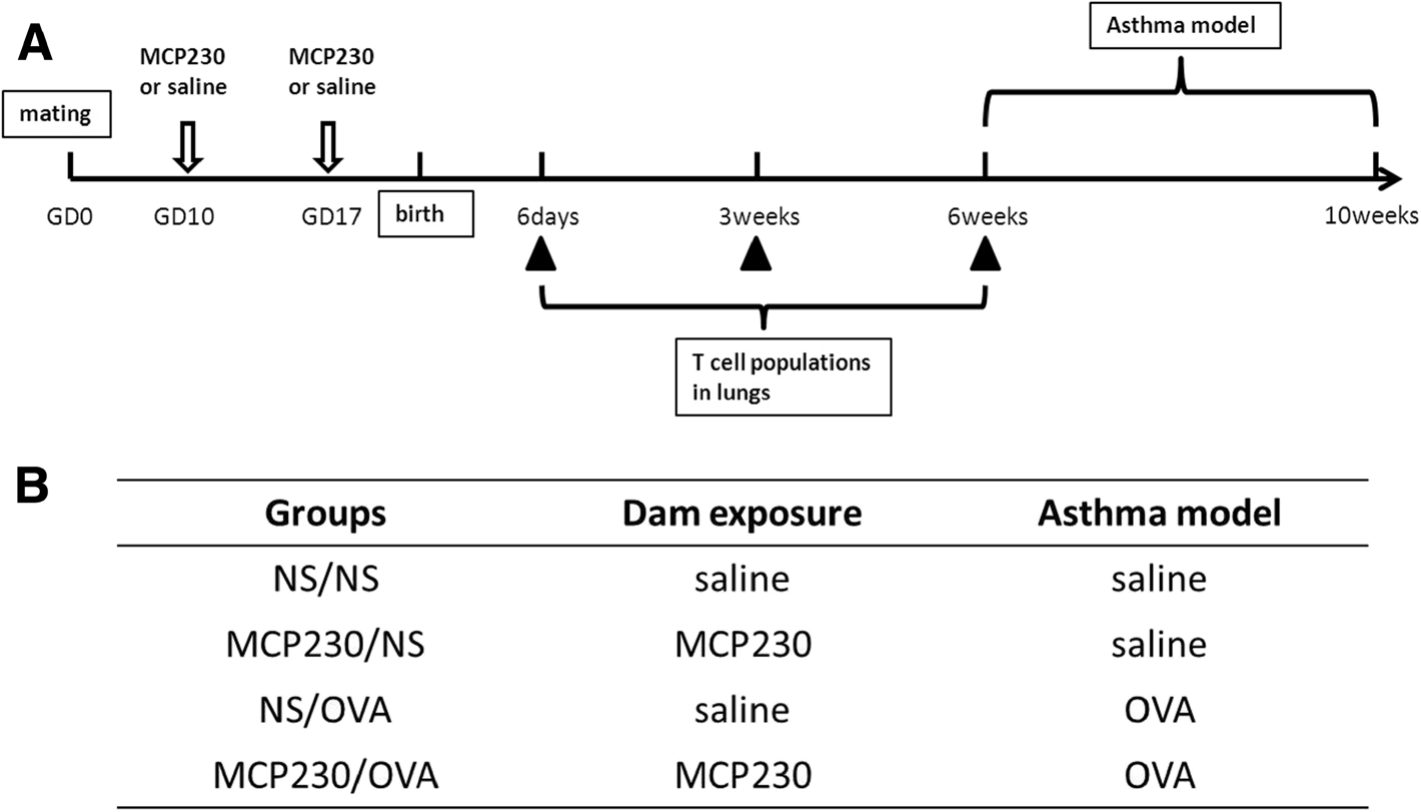
**The experimental protocol and groups. (A)** MCP230 or vehicle (NS) was administered to pregnant dams on gestational day (GD) 10 and 17. Pulmonary T cell populations were assessed in the offspring at six days, three weeks and six weeks of postnatal age. At six weeks of age, an OVA- model of asthma was established. **(B)** The experimental groups were shown in the table.

Eight-week-old C57/BL6 breeders were mated on day 0. On gestation day (GD) 10 and GD 17, pregnant mice were administered 50 μg MCP230 as previously described [14] (Figure 6). Briefly, MCP230 was suspended and sonicated in sterile saline to a final concentration of 1 mg/ml. Mice were administered MCP230 by oropharyngeal aspiration (O.A.). Control mice (NS) were administered with the same volume of saline. MCP230 was tested negative for lipooligosaccharide contamination.

### Assessment of oxidative stress in dam’s serum

Blood were collected from MCP230 or saline treated dams 3 days after the last exposure. After 30mins of clotting, the serum were isolated, quick frozen in liquid nitrogen, and stored at -80°C till further analysis. On the day of analysis, the serum were thawed and tested for 8 isoprostanes (8-IP), a marker of oxidative stress, using an ELISA kit (Cayman Chemical) as per manufacturer’s instruction.

### Assessment of pulmonary T cell populations

Lung samples were obtained from the infant mice at six days, three weeks and six weeks of postnatal age. A single-cell suspension of lung cells was prepared using a standardized protocol [29]. Briefly, lungs were perfused, excised, cut into small pieces, and incubated at 37°C for 1 h in RPMI 1640 media supplemented by 2% heat-inactivated FBS, 1 mg/ml collagenase I (Invitrogen), and 150 ng/ml DNase I (Sigma-Aldrich). After incubation, single cells were obtained by mashing the lung pieces through a 40 μm cell strainer (BD Biosciences). RBC were lysed using RBC lysis buffer (eBioscience), and the remaining cells were stimulated for 5 h with 5 ng/ml PMA and 500 ng/ml ionomycin (Sigma-Aldrich) in the presence of a protein transport inhibitor (GolgiPlug, BD Biosciences). Cells were then stained with the following antibodies: eFluor450-CD3, PerCP-CD4, PE-IFN-γ and PE-Cy7-IL-4, APC-IL-17A, FITC-Foxp3. Cell surface staining (i.e. CD3 and CD4) was performed before fixation/permeabilization. Fixable live/dead marker was used for dead cell exclusion. Cell staining was determined with a Canto II flow cytometer. Flow data were analyzed and plotted using FlowJo software. The gating strategy for T cell profile is illustrated in Additional file 1: Figure S1.

### Establishment of asthma model

When the offspring matured to adults (six weeks of age), an asthma model was established by ovalbumin (OVA) sensitization and challenge. Mice were divided into four groups (8-10 mice/group): maternal saline exposure/ saline challenge (NS/NS), maternal MCP230 exposure/ saline challenge (MCP230/NS), maternal saline exposure/ OVA challenge (NS/OVA), and maternal MCP230 exposure/ OVA challenge (MCP230/OVA). The NS/OVA and MCP230/OVA mice were immunized i.p. on days 0, 14 with 20 μg of OVA (Sigma-Aldrich, St. Louis, MO) emulsified in 100 μl Imject Alum (Pierce, Rockford, IL). On days 24, 25, 26, mice were challenged with aerosolized OVA (1% OVA in saline for 20 min). Age- and sex-matched mice that had been sensitized and challenged with saline at each time point were used as negative controls (NS/NS and MCP230/NS group).

### Pulmonary function test

Forty-eight hours after the final OVA challenge, respiratory mechanics was measured using an invasive method as previously described [30]. Briefly, anesthetized mice were intubated and mechanically ventilated by a computer controlled piston ventilator (flexiVent, Scireq). Mice were then challenged with an aerosolized bronchoconstrictor, methacholine (Mch; Sigma-Aldrich), at increasing doses: 0, 12.5, 25, 50, and 100 mg/ml). At each dose, lung resistance was calculated using the single compartment model.

### Bronchoalveolar lavage fluid (BALF) cellularity and cytokine measurement

After lung function was assayed a variety of samples were taken. Peripheral blood samples were obtained from the right ventricle and centrifuged for eight min at 800 g. The serum was collected for cytokine analysis. Bronchoalveolar lavage was performed by flushing lung with 1.0 ml PBS containing 2% BSA. Bronchoalveolar lavage fluid (BALF) and cells were isolating following centrifugation of lavage samples. BALF cells were counted and spun onto glass slides using a cytospin and stained using a Hema-3 staining kit (Fisher Scientific, Pittsburgh, PA). Differential cell counts were recorded based on the morphology and staining of the cells from a total of 200 cells per slide. Cytokine levels were measured from 25 μl of serum or cell-free BALF using a Milliplex mouse cytokine/chemokine assay kit (Millipore Corporation, Billerica, MA) according to the manufacturer’s instructions. Each sample was analyzed in duplicate on the Bio-plex system (Bio-Rad Laboratories, Hercules, CA). The sensitivity range of standards was ranging from 1.6 to 10,000 pg/ml. The concentrations of analytes in these assays were quantified using a standard curve, and a five-parameter logistic regression was performed to derive an equation that was then used to predict the concentration of the unknown samples. The following cytokines in BALF were assayed: IFN-γ, IL-4, IL-5, IL-10, IL-12p70, IL-13, IL-17, RANTES, KC, MCP-1 and MIP-1α. IL-17 and IL-12p70 in serum were also assayed. The data presented herein excluded any number below the range of sensitivity for the particular analyte.

### Lung histopathology

After the lavage was performed, the lungs were inflated by gentle infusion with HistoChoice tissue fixative (Amresco, Solon, OH) and isolated. The fixed lungs were then dehydrated, embedded in paraffin, and sectioned at 4 μm. Each lung section was stained with H&E and Periodic Acid-Schiff (PAS) to identify inflammation and mucus, respectively. Lung slides were digitalized with ImageScope (Aperio,Vista, CA) and mucus producing cells were quantified and expressed as positive staining cells per mm basement membrane.

### Statistical analysis

Each experiment was performed 2-3 times and the number of animals per group (n) is listed in the figure or table legend for each experiment. All data were analyzed using GraphPad InStat software (version 3.0). ANOVA was used to determine the levels of difference between all groups. Comparisons of all pairs were performed by Tukey-Kramer significant difference test. Values for all measurements are presented as means ± SEM. Values of *p* < 0.05 were considered statistically significant.

## Abbreviations

PM: Particulate matters; OVA: Ovalbumin; Th: T helper cells; Treg: T regulatory cells; EPFR-UFP: Ultrafine particle containing persistent Free Radicals; MCP230: EPFR-UFP containing 2-monochlorophenol that is formed by reaction with Cu(II)O containing fly-ash at 230°C; AHR: Airway hyperresponsiveness; Mch: Methacholine; BALF: Bronchoalveolar lavage fuild; GD: Gestational day; OA: Oropharyngeal aspiration; NS: Saline exposed or treated animals; IFN-γ: Interferon-gamma; SEM: Standard error of mean; CpG: Dinucleotide sequence cytosine linked to guanine via a phosphodiester bond in a linear DNA sequence.

## Competing interests

The authors have no competing interests.

## Authors’ contributions

PW designed and performed most experiments and wrote the manuscript draft. DY performed the flow cytometry experiments in asthma models, collected dam’s serums for 8-IP ELISA, and helped with manuscript preparation. JS assisted in pulmonary function studies, multiplex cytokine assays, and helped with manuscript preparation. HS assisted in manuscript preparation. SAC conceived and designed the study and revised the manuscript. All authors read and approved the final manuscript.

## Supplementary Material

Additional file 1: Figure S1 Flow cytometry gating strategy to identify T cell subsets. Lung cells were gated following this sequence: live cells, lymphocytes, CD3 + CD4+ T cells, and then IFN-γ + for Th1 cells, IL-4+ for Th2 cells, and IL-17+ for Th17 cells. Click here for file
