# Supplementary figures and images for: Maternal exposure to combustion generated PM inhibits pulmonary Th1 maturation and concomitantly enhances postnatal asthma development in offspring
Source: Part Fibre Toxicol. 2013 Jul 16;10:29. doi: 10.1186/1743-8977-10-29 (PMC3717277; doi:10.1186/1743-8977-10-29)

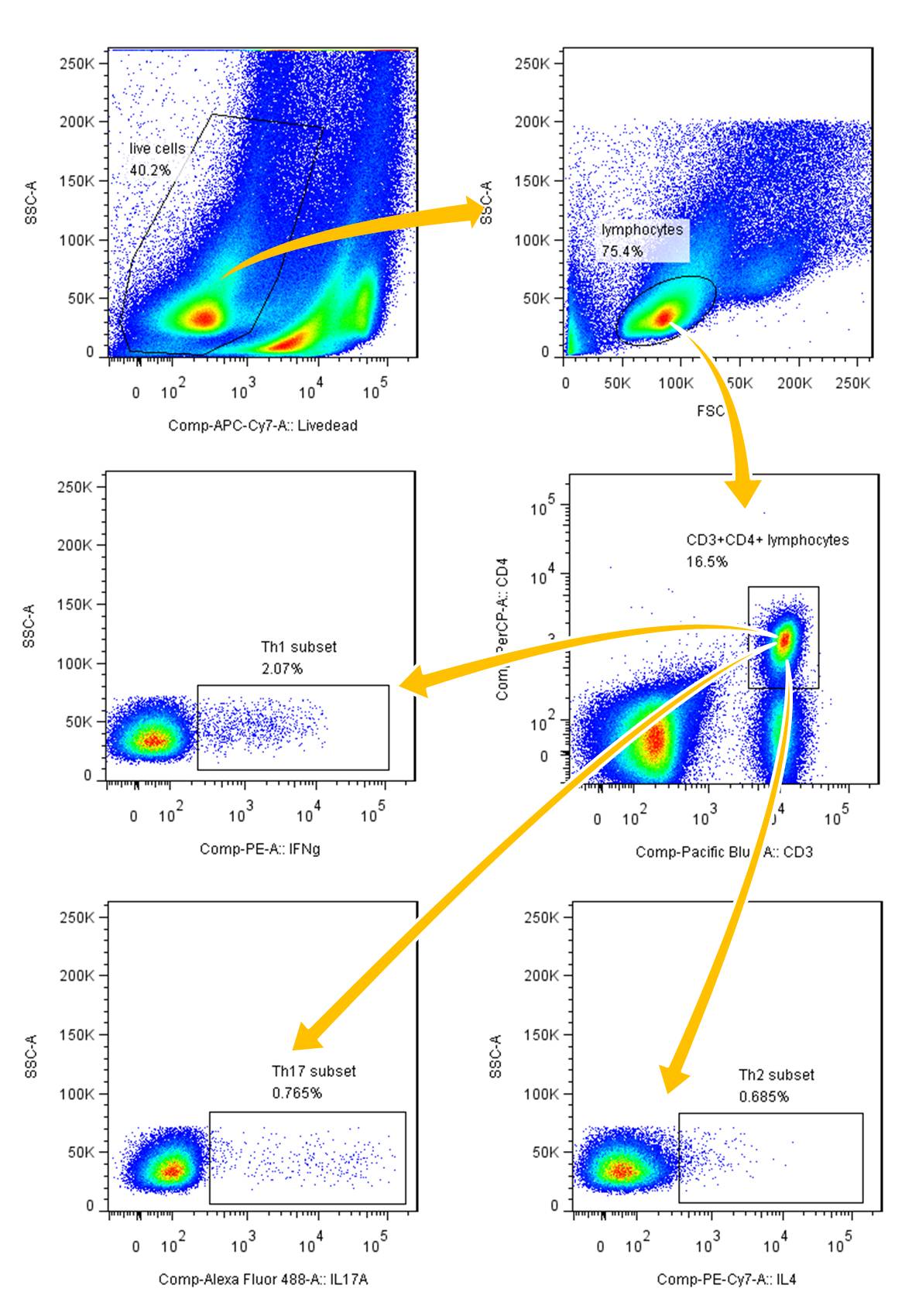

Supplement: Additional file 1: Figure S1 — Flow cytometry gating strategy to identify T cell subsets. Lung cells were gated following this sequence: live cells, lymphocytes, CD3 + CD4+ T cells, and then IFN-γ + for Th1 cells, IL-4+ for Th2 cells, and IL-17+ for Th17 cells. [file 1743-8977-10-29-S1.jpeg]
